# Differential effects of histone deacetylase inhibitors on cellular drug transporters and their implications for using epigenetic modifiers in combination chemotherapy

**DOI:** 10.18632/oncotarget.11561

**Published:** 2016-08-24

**Authors:** Benigno C. Valdez, Yang Li, David Murray, Jonathan E. Brammer, Yan Liu, Chitra Hosing, Yago Nieto, Richard E. Champlin, Borje S. Andersson

**Affiliations:** ^1^ Department of Stem Cell Transplantation and Cellular Therapy, University of Texas MD Anderson Cancer Center, Houston, Texas 77030, USA; ^2^ Department of Experimental Oncology, Cross Cancer Institute, Edmonton, Alberta T6G 1Z2, Canada

**Keywords:** HDAC inhibitors, drug transporter, combination therapy, lymphoma, pre-transplant regimen

## Abstract

HDAC inhibitors, DNA alkylators and nucleoside analogs are effective components of combination chemotherapy. To determine a possible mechanism of their synergism, we analyzed the effects of HDAC inhibitors on the expression of drug transporters which export DNA alkylators. Exposure of PEER lymphoma T-cells to 15 nM romidepsin (Rom) resulted in 40%-50% reduction in mRNA for the drug transporter MRP1 and up to ~500-fold increase in the MDR1 mRNA within 32-48 hrs. MRP1 protein levels concomitantly decreased while MDR1 increased. Other HDAC inhibitors − panobinostat, belinostat and suberoylanilide hydroxamic acid (SAHA) − had similar effects on these transporters. The protein level of MRP1 correlated with cellular resistance to busulfan and chlorambucil, and Rom exposure sensitized cells to these DNA alkylators. The decrease in MRP1 correlated with decreased cellular drug export activity, and increased level of MDR1 correlated with increased export of daunorubicin. A similar decrease in the level of MRP1 protein, and increase in MDR1, were observed when mononuclear cells derived from patients with T-cell malignancies were exposed to Rom. Decreased *MRP1* and increased *MDR1* expressions were also observed in blood mononuclear cells from lymphoma patients who received SAHA-containing chemotherapy in a clinical trial. This inhibitory effect of HDAC inhibitors on the expression of *MRP1* suggests that their synergism with DNA alkylating agents is partly due to decreased efflux of these alkylators. Our results further imply the possibility of antagonistic effects when HDAC inhibitors are combined with anthracyclines and other MDR1 drug ligands in chemotherapy.

## INTRODUCTION

Most chemotherapy drugs with different mechanisms of action are more effective when used in combination. Their combined cytotoxicity efficiently kills tumor cells, decreases the possibility of developing resistant cells, and provides patient safety when given at optimal doses. However, combined drugs may also have antagonistic effects, and this possibility underscores the importance of understanding their interactions.

Combination chemotherapy is a common part of pre-transplant conditioning regimens for patients undergoing hematopoietic stem cell transplantation (HSCT). DNA alkylating agents, nucleoside analogs, epigenetic modifiers, and proteasome inhibitors, among others, have been combined to minimize cancer burden prior to HSCT [1]. The synergistic effects of some of these drugs involve alteration of the chromatin structure, activation of the DNA-damage response, inhibition of DNA repair, disruption of mitochondrial membrane, and activation of apoptosis [1, 2]. Pre-clinical and clinical studies by our group showed the synergistic cytotoxicity of combined DNA alkylating agents busulfan (Bu) and melphalan (Mel), nucleoside analog gemcitabine (Gem), and an HDAC inhibitor in lymphoma cells [3–5]. We proposed a mechanism of their synergism that includes Gem-mediated inhibition of DNA synthesis resulting in histone modifications and opening of chromatin structure which allows the genomic DNA to be more accessible to the alkylating agents Bu and Mel [1, 2]. These alkylating drugs exacerbate DNA damage and activate apoptosis. Addition of an HDAC inhibitor (eg. suberoylanilide hydroxamic acid, SAHA) facilitates histone modifications and chromatin remodeling, in addition to possible increased expression of tumor suppressor genes, and further enhances Bu and Mel adduct formation.

In an effort to identify additional mechanisms of their synergism, we hypothesized that HDAC inhibitors may affect the expression of drug transporters, which are responsible for the efflux of structurally diverse anti-cancer drugs including DNA alkylators. The best characterized are the MRP1/ABCC1, MDR1/P-glycoprotein/ABCB, and BCRP/ABCG2; their expressions may correlate with multidrug resistance [6]. Although there is a significant overlap in their substrate specificity, MRP1 exports a broader range of therapeutic drugs including their glutathione (GSH)-conjugates [7–9].

Our present study shows that HDAC inhibitors down-regulate expression of the *MRP1* gene and up-regulate the *MDR1* gene. Since MRP1 exports GSH-conjugated DNA alkylators [7], a decrease in its protein level may contribute to the synergism of HDAC inhibitors and DNA alkylating agents. Conversely, HDAC inhibitors might antagonize the efficacy of anti-cancer drugs that are substrates for MDR1. These differential effects of HDAC inhibitors on the expression of drug transporters underscore the necessity for caution in combining these drugs with other chemotherapeutic agents.

## RESULTS

### HDAC inhibitors decrease the expression of *MRP1* but increase *MDR1* expression

**Figure 1:**
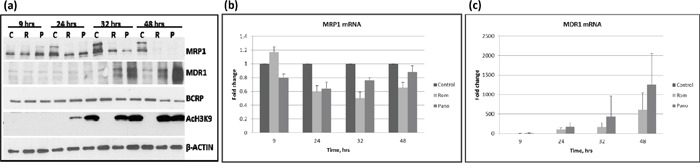
Kinetics of expression of MRP1, MDR1 and BCRP PEER cells were exposed to solvent (C, control), 15 nM romidepsin (R, Rom) or 150 nM panobinostat (P, Pano) and harvested after the indicated time (hrs). Total proteins and RNA were isolated and analyzed by Western blotting **a.** and quantitative real time-PCR **b** and **c.** respectively.

The HDAC inhibitor Romidepsin (Rom) has been reported to increase the expression of *MDR1* in patient mononuclear cells [10], but whether and how this drug affects the expression of other drug transporters is unknown. We, therefore, examined the effects of Rom and panobinostat (Pano) on the expression of three drug transporter genes – *MRP1*, *MDR1* and *BCRP* - at various time points in the PEER lymphoma cell line. Figure 1a shows similar effects of these two HDAC inhibitors; MRP1 protein levels started to decrease after 24-hr drug exposure and were almost eliminated after 48 hrs, while MDR1 protein levels started to increase after 32-hr drug exposure. On the other hand, BCRP protein levels slightly decreased after 48 hrs. Acetylation of histone 3 at Lys 9 (AcH3K9) started to increase after 24 hrs, suggesting the efficacy of Rom and Pano in inhibiting histone deacetylation. To determine if the effects of Rom and Pano on the expression of MRP1 and MDR1 were manifested at the transcription level, quantitative real-time PCR was performed. Figure 1b shows ~40% and ~50% decrease in the mRNA level of MRP1 after 24- and 32-hr Rom exposure, respectively; some recovery was apparent after 48 hrs. Maximum effect of Pano on the MRP1 mRNA was observed after 24 hrs and transcript levels started to recover after 32 hrs (Figure 1b). The mRNA level of MDR1 continued to increase from 24 to 48 hrs in the presence of either drug (Figure 1c).

**Figure 2:**
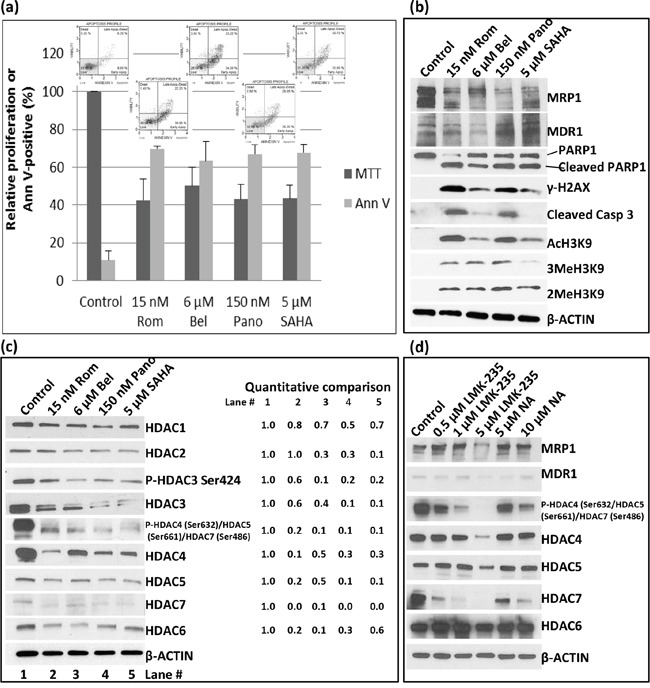
Effects of various histone deacetylase inhibitors on cell proliferation, apoptosis activation and histone modifications PEER cells were exposed to drugs for 48 hrs and analyzed for proliferation using the MTT assay **a.** Activation of apoptosis was determined using the Annexin V (Ann V; flow cytometry dot plots showing apoptosis profile for each treatment are on top of the bar graphs) assay (a) and Western blotting **b.** Modifications of histone 3 (b) and changes in the level of different histone deacetylases **c.** were determined by Western blotting. Quantitative analysis of the bands was done by normalizing the signals to β-ACTIN and calculating the ratio relative to the control (left side of panel c). **d.** Cells were exposed to HDAC Class II selective inhibitors LMK-235 or Nexturastat A (NA) at the indicated concentrations for 48 hrs and analyzed by Western blotting. Rom, romidepsin; Bel, belinostat, Pano, panobinostat; SAHA, suberoylanilide hydroxamic acid.

SAHA, an HDAC inhibitor, is a commonly used anti-neoplastic agent [11]. We, therefore, sought to determine if SAHA and belinostat (Bel) would have similar effects on the expression of *MRP1* and *MDR1* as Rom and Pano. We used drug concentrations approximately equivalent to their IC_50_ in the MTT assay (Figure 2a). At these concentrations apoptosis was activated as suggested by ~60% Annexin V positivity (Figure 2a) and cleavage of PARP1 and caspase 3 (Figure 2b). Again, MRP1 protein levels decreased in cells exposed to these HDAC inhibitors; MDR1 increased except in cells exposed to Bel (Figure 2b). DNA-damage response was activated as shown by increased phosphorylation of H2AX (Figure 2b). All four drugs inhibited histone deacetylase activity as suggested by increased levels of AcH3K9 with a corresponding increase in the methylation of histone 3 (Figure 2b). Additional Western blot analysis suggests that the observed increase in the level of AcH3K9 might be due to a decrease in the level of Class I and Class II histone deacetylases (Figure 2c). The phosphorylation of HDACs 3, 4, 5 and 7, which decreased in the presence of Rom, Bel, Pano and SAHA may also regulate the expression of *MRP1* and *MDR1* genes. Our data suggest that these HDAC inhibitors decrease expression of Class I and II HDAC with a concomitant increase in acetylated histone 3 and a corresponding down-regulation of *MRP1* but up-regulation of *MDR1*.

The observed down-regulation of HDAC4, -5, -6 and -7, which all belong to Class II, prompted us to determine if Class II selective inhibitors would similarly inhibit the expression of *MRP1* and up-regulate the expression of *MDR1*. Western blot analysis shows a decrease in the level of MRP1 protein in cells exposed to 5 μM LMK-235, a selective inhibitor of HDAC4 and -5, but not in cells exposed to 0.5- 1 μM LMK-235 nor 5-10 μM Nexturastat A (NA), a selective inhibitor of HDAC6; the level of MDR1 protein was not significantly affected (Figure 2d). The phosphorylation status of HDAC4, 5 and 7 dramatically decreased in cells exposed to 0.5 – 5 μM LMK-235 and 5 -10 μM NA but their respective level of total protein significantly changed only at 5 μM LMK-235 (Figure 2d). The level of HDAC6 was not significantly affected by either drug. These results suggest that among the members of Class II group HDAC4, -5 and -7 but not HDAC6 may be involved in the regulation of the *MRP1* gene expression but not *MDR1*.

### HDAC inhibitors decrease MRP1 activity

**Figure 3:**
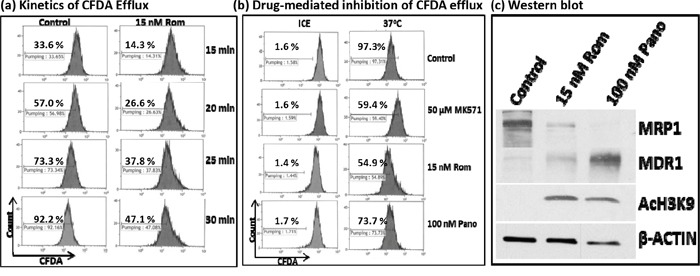
Functional assay of MRP1 protein PEER cells were exposed to solvent (Control) or 15 nM romidepsin (Rom) for 48 hrs, harvested, incubated with the MRP1 substrate CFDA for 20 min, shifted to 37°C, and analyzed for efflux of CFDA at the indicated time point by flow cytometry **a.** To determine drug-mediated inhibition of CFDA efflux, cells were exposed to 15 nM Rom or 100 nM Pano (panobinostat) for 48 hrs or to the MRP1 inhibitor MK571 (50 μM) for 30 min prior to the efflux assay. CFDA efflux was performed for 30 min **b.** “Count” refers to the number of live (i.e., PI-negative) cells. Western blot analysis confirmed the 48-hr drug-mediated decrease in MRP1 protein level **c.**

At the molecular level, RT-PCR and Western blot analyses showed decreased levels of MRP1 mRNA and protein. We next sought to determine if cells exposed to HDAC inhibitors would have a decreased functional ability to export specific drugs using flow cytometry. We initially analyzed the kinetics of efflux of CFDA, a known substrate for MRP1 [12], in PEER cells previously exposed to solvent or 15 nM Rom as described in Figure 3. Cells were kept on ice and allowed to take up CFDA for 20 min, then shifted to 37°C to allow efflux of the fluorescent substrate for various periods of time. The control cells exported ~33% – 73% of CFDA after 15-25 min, increasing to ~92% efflux after 30 min (Figure 3a). On the other hand, cells previously exposed to Rom showed only ~47% efflux of CFDA after 30 min (Figure 3a), suggesting almost 50% inhibition of the CFDA efflux in Rom-treated cells. To confirm if efflux of CFDA was mediated by MRP1, cells were exposed to MK571, a known inhibitor of MRP1 [13]; efflux was indeed inhibited by ~40% compared to control cells (Figure 3b). Rom (15 nM) and 100 nM Pano decreased CFDA efflux from ~97% (Control) to ~55% and ~74%, respectively. Again, the drug-mediated decrease in MRP1 protein level was confirmed by Western blotting (Figure 3c). It is not clear why exposing PEER cells to 100 nM Pano almost eliminated MRP1 protein (Figure 3c) but inhibited CFDA efflux by only ~23% (Figure 3b). Overall, these results show strong correlation of decreased MRP1 mRNA/protein levels and transport activity in cells exposed to HDAC inhibitors.

### HDAC inhibitors increase MDR1 activity

**Figure 4:**
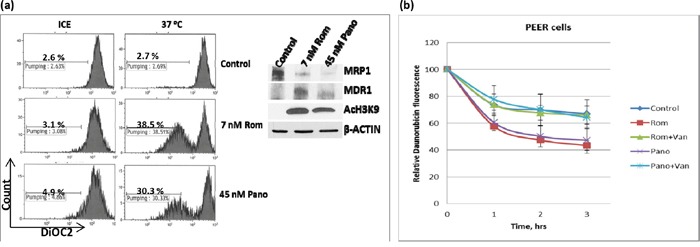
Functional assay of MDR1 protein **a.** U937 cells were exposed to solvent (Control), 7 nM romidepsin (Rom) or 45 nM panobinostat (Pano) for 48 hrs, harvested, incubated with MDR1 substrate DiOC_2_(3) for 20 min, shifted to 37°C for 45 min and analyzed for efflux of DiOC_2_(3) by flow cytometry as described under Materials and Methods. “Count” refers to the number of live (i.e., DAPI-negative) cells. Total protein extracts were analyzed by Western blotting (inset) to determine changes in the level of MDR1 protein. **b.** A similar analysis was performed using daunorubicin as a fluorescent substrate for MDR1 in the absence or presence of 2 mM orthovanadate (Van), an MDR1 inhibitor.

We similarly analyzed the effects of HDAC inhibitors on the drug transport activity of MDR1 in U937 cells by flow cytometry using DiOC_2_(3), a known substrate for MDR1 [14]. Exposure of cells to 7 nM Rom or 45 nM Pano decreased MRP1 but increased MDR1 protein levels (Figure 4a, inset). Efflux of DiOC_2_(3) from cells kept on ice during the efflux assay was not significant for either control or drug-treated cells; shifting to 37°C showed almost negligible efflux of DiOC_2_(3) from the control cells but efflux increased to ~39% and ~30% for cells pre-exposed to Rom and Pano, respectively (Figure 4a). The observed efflux activity correlated with the level of MDR1 protein as shown by Western blotting.

Since MDR1 is known to catalyze export of anthracyclines such as the anti-neoplastic drugs daunorubicin and doxorubicin [15], we determined if daunorubicin efflux would be increased in U937 cells pre-exposed to Rom or Pano. The decrease in cellular daunorubicin fluorescence, as measured by flow cytometry, was used as an indicator of drug efflux. Cells were allowed to take up daunorubicin for 1.5 hrs at 37°C, washed and incubated in drug-free culture medium. Approximately 30% efflux of daunorubicin from the control cells occurred after 3 hrs. Daunorubicin efflux increased to ~58% and ~55% in cells pre-exposed to Rom and Pano, respectively (Figure 4b). Addition of orthovanadate, a known inhibitor of ATPase-dependent drug transporters [16, 17], abrogated the Rom- and Pano-mediated increase in efflux of daunorubicin (Figure 4b). Overall, these results show that HDAC inhibitors increase the drug transport activity of MDR1, consistent with increased MDR1 mRNA and protein levels (Figure 1).

### The level of expression of *MRP1* correlates with cellular sensitivity to DNA alkylating agents

**Figure 5:**
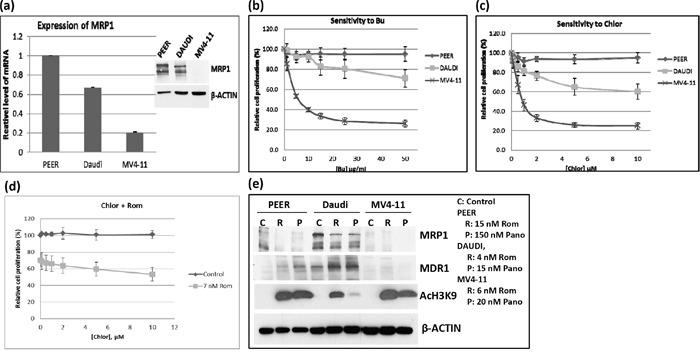
Correlation between the level of MRP1 protein and cell sensitivity to DNA alkylating agents **a.** The expression of MRP1 in PEER, Daudi and MV4-11 cells was determined by quantitative real time-PCR (mRNA level) and Western blotting (protein level, inset). **b, c.** The three cell lines were exposed to various concentrations of busulfan (Bu) or chlorambucil (Chlor) for 48 hrs and analyzed for cell proliferation using the MTT assay. **d.** PEER cells were exposed to Chlor with or without 7 nM Rom for 48 hrs and analyzed by the MTT assay. **e.** Cells were exposed to HDAC inhibitors (inset: drug concentrations) for 48 hrs and total protein extracts were analyzed by Western blotting.

DNA alkylating agents such as Bu and Mel exert synergistic cytotoxicity when combined with HDAC inhibitors in pre-clinical and clinical studies [3–5]. Moreover, GSH-conjugated alkylators are known to be exported from cells via the MRP1 drug transporter [7]. Based on these previous studies and on the observed down-regulation of *MRP1* at the mRNA, protein and functional levels, we hypothesized that treatment of cells with HDAC inhibitors may result in inhibition of the efflux of GSH-conjugated alkylators. To test this hypothesis, we first examined the correlation between the basal level of MRP1 and the cellular sensitivity to DNA alkylating agents of PEER (T-cell leukemia), Daudi (B-cell lymphoma) and MV4-11 (AML) cell lines. The level of expression of *MRP1* was highest in PEER and lowest in MV4-11 cells (Figure 5a). Dose-response analysis showed highest resistance of PEER cells to two DNA alkylating agents, busulfan and chlorambucil, and least resistance of MV4-11 cells (Figure 5b and 5c). PEER cells became more sensitive to chlorambucil (Figure 5d) and busulfan (data not shown) in the presence of Rom. Whereas proliferation of PEER cells was not significantly affected by 0 - 10 μM chlorambucil (slope of the dose-response line = −0.05), a steeper slope of -1.6 was obtained in the presence of 7 nM Rom, suggesting a Rom-mediated decrease in resistance of PEER cells to chlorambucil (Figure 5d). Again, exposure of all three cell lines to Rom or Pano decreased the level of MRP1 protein (Figure 5e). The observed correlation between DNA alkylator sensitivity and the level of MRP1 protein is consistent with the previous report that MRP1 exports DNA alkylators from cells [7, 8], and suggests that the synergism of these drugs with HDAC inhibitors is partly due to down-regulation of the expression of *MRP1*.

### HDAC inhibitors down-regulate *MRP1* and up-regulate *MDR1* in patient-derived cell samples

**Figure 6:**
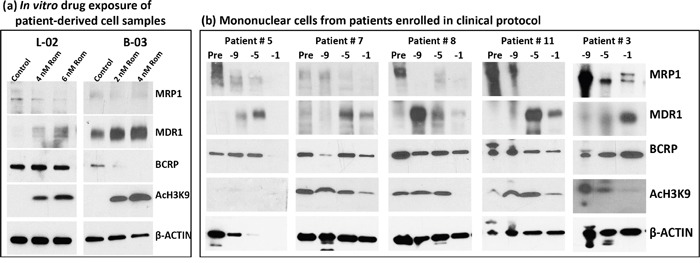
Effects of HDAC inhibitors on patient-derived cell samples **a.** Mononuclear cells from blood of patients with peripheral T-cell lymphoma (L-02) and T-cell prolymphocytic leukemia (B-03) were isolated and exposed to the indicated concentrations of romidepsin (Rom) for 48 hrs, and total protein extracts were analyzed by Western blotting. **b.** Mononuclear cells from patients enrolled in clinical trial NCT01983969 were isolated and analyzed by Western blotting. These patients received a pretransplant conditioning regimen that included gemcitabine, busulfan, melphalan, azacytidine and SAHA. SAHA (Vorinostat) was given on days −11 to −3 prior to hematopoietic stem cell transplantation. Baseline samples (Pre) were taken before the conditioning therapy and treated samples at 9, 5, 1 day(s) prior to transplant. Sampling prior to chemotherapy for patient #3 was not performed for logistical reasons.

To determine the potential clinical significance of our cell line studies, we exposed mononuclear cells isolated from the peripheral blood of patients with T-cell malignancies (patient characteristics are shown in Table 1, Supplementary Materials) to Rom and analyzed them by Western blotting. The MRP1 protein levels decreased in a Rom concentration-dependent manner while the MDR1 protein levels increased in both patient samples (Figure 6a). While the level of BCRP did not change in sample L-02, it significantly decreased in sample B-03 in the presence of Rom (Figure 6a). We extended this study to an analysis of mononuclear cells from patients enrolled in a clinical trial evaluating SAHA, azacytidine, Gem, Bu and Mel as a pre-transplant conditioning regimen [18]. Patients received SAHA starting at day −11 prior to stem cell transplantation. Figure 6b shows a decrease in the MRP1 protein level from day −9 to day −1 relative to its baseline level (pre-chemotherapy) while the MDR1 protein level increased in all 5 patient samples and the BCRP level was not greatly affected. These results are consistent with our *in vitro* cell line and patient cell sample studies (Figures 1 and 6a), suggesting that HDAC inhibitors differentially affect the expression of *MRP1* and *MDR1* both in *in vitro* and *in vivo* settings.

## DISCUSSION

We previously demonstrated the synergistic cytotoxicity of DNA alkylating agents, nucleoside analogs and HDAC inhibitors in various experimental models [3–5]. Here, we present a novel mechanism of their synergism in established cell lines and patient-derived cell samples. HDAC inhibitors were seen to down-regulate the expression of *MRP1*, resulting in decreased MRP1 protein levels and drug transport activity (Figures 1, 3). Since MRP1 is known to pump GSH-conjugated DNA alkylators out of cells [7], decreased MRP1 activity may cause cellular accumulation of DNA alkylating agents and thus enhanced cytotoxicity. This inference is consistent with (1) a strong inverse correlation between the level of MRP1 protein and sensitivity to alkylating agents; cells with high levels of MRP1 are more resistant to Bu and chlorambucil, but this phenotype is reversed when cells are exposed to Rom (Figure 5), and (2) feedback inhibition of glutathione S-transferases by the GSH-DNA alkylator conjugates, leading to increased intracellular concentrations of free alkylating agents [19].

The inhibitory effect of HDAC inhibitors on *MRP1* expression is apparent at the transcription level (Figure 1b). Post-transcriptional regulation(s), however, may also contribute to these effects, as suggested by a greater decrease in the MRP1 protein compared with its mRNA levels (Figure 1). Changes in the acetylation status of proteins involved in the translation of MRP1 mRNA, and possibly MRP1 protein itself, may contribute to the reduction of MRP1 levels. Rom and Pano down-regulate *MRP1* expression within 24 hrs of drug exposure as indicated by decreased mRNA levels. These changes coincide with histone modifications (Figure 1a), which may inhibit or enhance transcription [20, 21]. Acetylation and methylation of histone 3 are both enhanced in PEER cells exposed to Rom, Bel, Pano and SAHA (Figure 2b); acetylation is partly due to decreased protein levels of HDACs 3, 4 and 6 (Figure 2c), and methylation is most likely due to the known functional interaction between histone methyltransferases and deacetylases [22]. It remains to be determined which histone modifications contribute to the inhibition of *MRP1* expression.

Similar results were observed in patient-derived cell samples. Exposure of cells, obtained from patients with peripheral T-cell lymphoma and T-cell prolymphocytic leukemia, to Rom decreased the MRP1 protein level (Figure 6a). Mononuclear cells isolated from patients treated with a SAHA-containing chemotherapy regimen also showed decreased MRP1 relative to samples taken prior to chemotherapy (Figure 6b). These data show that both *in vitro* and *in vivo* exposures of cells to HDAC inhibitors resulted in down-regulation of the *MRP1* gene.

While HDAC inhibitors down-regulate the *MRP1* gene, they up-regulate the expression of the *MDR1* gene. This effect is also apparent at the transcription level within 24 hrs of drug exposure (Figure 1c) with a concomitant increase in protein levels and drug transport activity of MDR1 (Figure 1a and 4c). Similar results were obtained in established cell lines and patient-derived cell samples, suggesting general differential effects of HDAC inhibitors on the expression of drug transporter genes. The mechanisms underlying these differential effects remain unknown, but may be related to differences in the site-specific acetylation/methylation of histones. For example, acetylation and monomethylation of histone 3 at Lys 9 activate gene transcription whereas di- and tri-methylation of the same residue results in repression [23–25]. Changes in histone modifications may also affect expression of activators and repressors of gene transcription, as well as mediating a switch between transcription permissive and repressive chromatin structures [20].

Our study provides a potentially novel approach to reverse drug resistance in patients. While few inhibitors which directly bind to drug transporters such as verapamil, phenothiazine and cyclosporine D derivatives [26] have been unsuccessful in overcoming drug resistance in the clinic albeit their efficacy in laboratory models, the use of HDAC inhibitors to down-regulate *MRP1* expression occurs at the gene level. In fact, our clinical study shows SAHA-mediated down-regulation of *MRP1* expression (Figure 6b). Although a well-designed and controlled clinical trial is needed to unequivocally prove the role of HDAC inhibitors in the reversal of MRP1-mediated drug resistance in the clinic, our laboratory and clinical data suggest its possibility.

In summary, our results present potentially critical implications for combining HDAC inhibitors with other chemotherapy agents. Combination of HDAC inhibitors with DNA alkylators and other MRP1 substrates is expected to result in synergistic and more efficacious outcomes. However, the increased MDR1 expression induced by HDACi may result in reduced anticancer activity of MDR1 substrates, such as anthracyclines, vinca alkaloids and steroids. Our data seem timely and relevant in light of a currently ongoing clinical trial (NCT01280526), testing the value of combined romidepsin and CHOP (cyclophosphamide, adriamycin, vincristine, and prednisone). In particular, efflux of adriamycin, vincristine and prednisone may increase with HDAC inhibitor-mediated up-regulation of the *MDR1* gene [6]. Various drug combinations are used in pre-transplant conditioning prior to hematopoietic stem cell transplantation, and our study highlights the importance of understanding the mechanism(s) of drug interactions to achieve a more efficacious cytotoxicity to tumor cells.

## MATERIALS AND METHODS

### Cells and drugs

PEER is a cell line originally isolated and established from a patient with T-cell acute lymphoblastic leukemia [27], Daudi (ATCC, Manassas, VA) is a B-lymphoblast cell line, U937 (ATCC) is a cell line originally established from a patient with histiocytic lymphoma [28], and MV4-11 (ATCC) is a biphenotypic B myelomonocytic leukemia cell line. Cells were grown in RPMI 1640 medium (Mediatech, Manassas, VA) supplemented with 10% heat-inactivated fetal bovine serum (Sigma-Aldrich, St Louis, MO) and 100 U/ml penicillin and 100 μg/ml streptomycin (Mediatech) at 37°C in a fully humidified atmosphere of 5% CO_2_ in air. Romidepsin (Rom), panobinostat (Pano), belinostat (Bel), SAHA, LMK-235 and Nexturastat A were purchased from Selleck Chemicals (Houston, TX) and dissolved in dimethyl sulfoxide (DMSO) to prepare stock solutions.

### Patient samples

Two lymphocytic leukemia cell samples (L-02 and B-03) were isolated from patient peripheral blood using lymphocyte separation medium (Mediatech) and incubated in suspension in the same RPMI 1640 medium described above. Patient L-02 had leukemic peripheral T-cell lymphoma, whereas patient B-03 had T-cell prolymphocytic leukemia in the setting of Li-Fraummeni syndrome (congenital p53 deficiency). Cells were exposed to DMSO or DMSO/romidepsin for 48 hrs and analyzed by Western blotting.

Mononuclear cells from peripheral blood of patients enrolled in clinical trial NCT01983969 (PI: Dr. Yago Nieto) were isolated using lymphocyte separation medium (Mediatech), lysed and analyzed by Western blotting.

All patient samples were collected after obtaining written informed consent, and all studies using these patient samples were performed under a protocol approved by the Institutional Review Board of the University of Texas MD Anderson Cancer Center, in accordance with the Declaration of Helsinki.

### Western blot analysis

Cells were exposed continuously to drugs for 48 hrs, harvested, washed with cold phosphate-buffered saline (PBS), and lysed with lysis buffer (Cell Signaling Technology, Danvers, MA). Cell extracts were analyzed for protein concentrations using a BCA Protein Assay kit (ThermoFisher Scientific, Rockford, IL), and immunostaining by Western blot was done as previously described [28]. The antibodies used in this study are listed in Table 2 (Supplementary Materials).

### Quantitative real-time PCR analysis

Total RNA was purified using the RNeasy mini kit (Qiagen, Valencia, CA) and complementary DNA was synthesized using the High Capacity cDNA Reverse Transcription Kit with RNase Inhibitor (Applied Biosystems, Foster City, CA). Gene expression was determined by RT-PCR analysis using the iTAQ Universal SYBR Green Supermix (Bio-Rad Lab, Hercules, CA) as described [29]. The primers used are listed in Table 3 (Supplementary Materials).

### Cell proliferation and cell death assays

Cell proliferation assays were done in triplicate using 96-well plates. Cells (4 × 10^4^) in 100 μl medium were incubated with or without the test HDAC-inhibiting drug at 37°C, 5% CO_2_ for 48 hrs and analyzed for proliferation using the 3-(4, 5-dimethylthiazol-2-yl)-2, 5-diphenyl tetrazolium bromide (MTT) assay as previously described [29]. Programmed cell death was determined using the Annexin V assay which measures the externalization of phosphatidylserine by flow cytometry as described [30].

### Functional assays for MRP1 and MDR1

5-Carboxyfluorescein diacetate (CFDA from Sigma-Aldrich) and 3, 3′-diethyloxacarbocyanine iodide (DiOC_2_(3) from EMD Millipore, Billerica, MA) were used as fluorescent substrates to assay the MRP1 and MDR1 transport activity, respectively, by flow cytometry. Cells were exposed to solvent or HDAC inhibitor for 48 hrs at 37°C, harvested and resuspended in fresh RPMI 1640 medium containing either 1 μM CFDA or 0.5 μg/ml DiOC_2_(3) and kept on ice for 20 min to allow their uptake/influx. Cells were centrifuged, washed with cold PBS and resuspended in 1 ml RPMI 1640 medium. Each cell suspension was divided into two 0.5 ml aliquots; one set was kept on ice and the other set was shifted to 37°C for up to 30 min (CFDA) or 45 min (DiOC_2_(3)) to allow temperature-dependent substrate efflux. Cells were centrifuged again at 4°C, resuspended in 0.4 ml PBS containing 0.1 μg/ml propidium iodide (PI), and analyzed by flow cytometry using excitation/emission wavelength of 488/525 nm for CFDA and DiOC2(3), and 488/617 nm for PI. Live and dead cells were differentiated by inclusion of PI, and only live cells were analyzed.

The above procedure was modified when daunorubicin (Selleck Chemicals, Houston, TX) was used as a substrate for the efflux assay. Cells were exposed to solvent or HDAC inhibitor for 48 hrs. Daunorubicin (2 μM) was added to the cell suspension and incubated for 1.5 hrs at 37°C, 5% CO_2_ to allow its uptake. Cells were pelleted, washed with PBS (room temperature) and resuspended in warm RPMI 1640 medium. Cells were further incubated at 37°C, 5% CO_2_ to allow daunorubicin efflux. Sample aliquots were taken after 0, 1, 2 and 3 hrs. Cells were centrifuged, resuspended in 0.4 ml PBS containing 2 μg/ml 4′, 6-diamidino-2-phenylindole (DAPI) and analyzed by flow cytometry using excitation/emission wavelength of 488/575 nm for daunorubicin and 405/461 nm for DAPI. Live and dead cells were differentiated by inclusion of DAPI and only live cells were analyzed.

To inhibit efflux of daunorubicin, 2 mM sodium orthovanadate (Selleck Chemicals) was added 1 hr after the addition of daunorubicin and incubated for another 30 min. Efflux assay was performed as described above in the presence of 2 mM sodium orthovanadate.

## SUPPLEMENTARY TABLES


