# Supplementary material for: Differential effects of histone deacetylase inhibitors on cellular drug transporters and their implications for using epigenetic modifiers in combination chemotherapy
Source: Oncotarget. 2016 Aug 24;7(39):63829–38. doi: 10.18632/oncotarget.11561 (PMC5325407; doi:10.18632/oncotarget.11561)
Supplement: Supplementary file 1 [file oncotarget-07-63829-s001.pdf]

Differential effects of histone deacetylase inhibitors on cellular drug transporters and their implications for using epigenetic modifiers in combination chemotherapy

SUPPLEMENTARY TABLES

Supplementary Table S1: Characteristics of patients whose cell samples were used in the study

Patient	Age, yrs	Gender	Race	Diagnosis
L-02	61	M	African-American	Peripheral T-cell lymphoma
B-03	37	M	Caucasian	T-cell prolymphocytic leukemia
3	25	F	Unknown	Anaplastic large cell lymphoma
5	57	F	Hispanic or Latino	non-Hodgkin's lymphoma
7	41	M	African-American	Hodgkin's lymphoma
8	38	M	Hispanic or Latino	Hodgkin's lymphoma
11	46	M	Unknown	Hodgkin's lymphoma

Supplementary Table S2: List of primary antibodies, their sources and dilutions

Antigen	Source/Cat. #	Clone type*	Dilution**
AcH3K9	Active Motif/39137	pAb	2500
BCRP	Abcam/108312	mAb	2500
Cleaved Caspase 3	Cell Signaling/9661	pAb	2500
HDAC1	Cell Signaling/5356	mAb	2500
HDAC2	Cell Signaling/5113	mAb	3000
HDAC3	Cell Signaling/3949	mAb	2500
P-HDAC3 Ser424	Cell Signaling/3815	pAb	2000
HDAC4	Cell Signaling/5392	mAb	2500
P-HDAC4(Ser632)/ HDAC5(Ser661)/ HDAC7(Ser486)	Cell Signaling/3424	pAb	2000
HDAC5	Cell Signaling/2082	pAb	2500
HDAC6	Cell Signaling/7558	mAb- R	2500
HDAC7	Cell Signaling/2882	pAb	1500
MDR1	Abcam/170904	mAb	2000
MRP1	Abcam/24102	mAb	2000
PARP1	Santa Cruz/SC8007	mAb	1000
β-ACTIN	Sigma/A5316	mAb	6000
γ-H2AX	EMD Millipore/05-636	mAb	3000
2MeH3K9	Active Motif/39683	mAb	2500
3MeH3K9	Active Motif/39161	pAb	3500

* pAb: polyclonal antibody; used anti-rabbit IgG for secondary antibody from Bio-Rad Lab mAb: monoclonal antibody; used anti-mouse IgG (or anti-rabbit IgG as indicated - R) for secondary antibody from Bio-Rad Lab
**Fold dilution in PBS with 0.05% Tween 20

Supplementary Table S3: Primers for real-time PCR analysis

Gene	Forward	Reverse	Annealing Temp (°C)
GAPDH	5'-CAACAGCCTCAAGATCATCAGC-3'	5-'TCCTAGACGGCAGGTCAGGTC-3'	60
MRP1	5'-CGGAAACCATCCACGACCCTAATC-3'	5'-ACCTCCTCATTCGCATCCACCTTGG-3'	60
MDR1	5'-GCCTGGCAGCTGGAAGACAAATACAC AAAATT-3'	5-'CAGACAGCAGCTGACAGTCCAAGAA CAGGACT-3'	60
